# Treatment Outcomes and Toxicity of Cisplatin–Etoposide-Based Regimens and Carboplatin–Paclitaxel as Adjuvant Chemotherapy for Ovarian Adult Granulosa Cell Tumors: A Multicenter Retrospective Cohort Study

**DOI:** 10.3390/cancers18182910

**Published:** 2026-09-09

**Authors:** Aslı Geçgel, Mehmet Uzun, Mehmet Burak Kaya, Seval Ay Ersoy, Tuba Uğur Tuzcu, Tuğba Kaya, Yiğit Efe Sürgen, Ekin Kavvasoğlu Durmuş, Canan Kelten Talu, Zafer Arık, Osman Sütçüoğlu, Nuri Yıldırım, Erdem Göker, Sercan Ön

**Affiliations:** 1 Department of Medical Oncology, Ege University Faculty of Medicine, 35100 Izmir, Türkiye; asli.gecgel@ege.edu.tr (A.G.); erdem.goker@ege.edu.tr (E.G.); 2 Department of Medical Oncology, Izmir Tepecik Training and Research Hospital, 35020 Izmir, Türkiye; mehmet.uzun@saglik.gov.tr; 3 Department of Medical Oncology, Hacettepe University Faculty of Medicine, 06230 Ankara, Türkiye; burak_kaya@hacettepe.edu.tr (M.B.K.); zafer.arik@hacettepe.edu.tr (Z.A.); 4 Department of Medical Oncology, Kartal Dr. Lutfi Kirdar City Hospital, 34865 Istanbul, Türkiye; seval.ayersoy@saglik.gov.tr (S.A.E.); tugba.kaya3@saglik.gov.tr (T.K.); 5 Department of Medical Oncology, Gazi University Faculty of Medicine, 06560 Ankara, Türkiye; tubaugurtuzcu@gazi.edu.tr (T.U.T.); osmansutcuoglu@gazi.edu.tr (O.S.); 6 Department of Internal Medicine, Ege University Faculty of Medicine, 35100 Izmir, Türkiye; yigit.efe.surgen@ege.edu.tr; 7 Department of Internal Medicine, Tepecik Training and Research Hospital, 35020 Izmir, Türkiye; ekin.kavvasoglu@saglik.gov.tr; 8 Department of Medical Pathology, Tepecik Training and Research Hospital, 35020 Izmir, Türkiye; esracanankelten.talu@sbu.edu.tr; 9 Department of Obstetrics and Gynecology, Ege University Faculty of Medicine, 35100 Izmir, Türkiye; nuri.yildirim@ege.edu.tr

**Keywords:** adult granulosa cell tumor, adjuvant chemotherapy, platinum-based chemotherapy, carboplatin–paclitaxel, bleomycin–etoposide–cisplatin, disease-free survival, toxicity, multicenter study

## Abstract

**Background/Objectives:** Adult granulosa cell tumors (AGCTs) of the ovary are rare sex cord-stromal malignancies characterized by an indolent clinical course and a persistent risk of late recurrence. Although adjuvant chemotherapy is considered for selected patients with higher-risk disease, the optimal platinum-based regimen remains uncertain because comparative evidence is limited. This study compared disease-free survival (DFS), treatment delivery, and toxicity between bleomycin–etoposide–cisplatin/etoposide–cisplatin (BEP/EP) and carboplatin–paclitaxel (CP) in the postoperative adjuvant setting. **Methods:** This retrospective, multicenter, nonrandomized comparative cohort study included 58 patients with AGCTs who underwent primary surgery and received adjuvant platinum-based chemotherapy at five tertiary oncology centers in Türkiye between 2010 and 2024. Patients received BEP/EP or CP according to institutional practice and physician discretion. The primary endpoint was disease-free survival (DFS), while secondary endpoints included treatment delivery and treatment-related toxicity. **Results:** A total of 58 patients were included, with 26 receiving BEP/EP and 32 receiving CP. Patients treated with CP were older, whereas fertility-sparing surgery was more common in the BEP/EP group. After a median follow-up of 75.5 months, 14 recurrences were observed; when deaths without prior recurrence were included according to the prespecified DFS definition, a total of 19 DFS events occurred. Median DFS was 77.2 months in the BEP/EP group and 126.2 months in the CP group. The estimated 5-year DFS rates were 75.5% and 83.5%, respectively, with no statistically significant difference between treatment groups (log-rank *p* = 0.296). In an exploratory multivariable Cox model restricted to age and treatment regimen, increasing age was associated with a higher risk of a DFS event (adjusted HR 1.046 per year, 95% CI 1.011–1.082; *p* = 0.010), whereas CP versus BEP/EP was not significantly associated with DFS (adjusted HR 0.426, 95% CI 0.167–1.083; *p* = 0.073). Treatment completion rates were numerically higher with CP (84.4% vs. 69.2%; *p* = 0.17). Peripheral neuropathy occurred more frequently with CP (*p* = 0.007), whereas nephrotoxicity occurred only in the BEP/EP group (*p* = 0.014). Pulmonary toxicity was observed only with bleomycin-containing treatment, and one patient developed secondary acute myeloid leukemia following BEP. **Conclusions:** No statistically significant difference in DFS was detected between BEP/EP and CP, while distinct toxicity patterns were observed. Given the retrospective design, limited sample size, and potential for treatment-selection bias and residual confounding, these findings should be considered exploratory and should not be interpreted as evidence of equivalent efficacy between the regimens.

## Simple Summary

Adult granulosa cell tumors of the ovary are rare malignancies, and the role of adjuvant chemotherapy after surgery remains uncertain. Bleomycin–etoposide–cisplatin has traditionally been used, while carboplatin–paclitaxel has increasingly been adopted as an alternative regimen. This multicenter retrospective study evaluated disease-free survival, treatment delivery, and toxicity in 58 patients treated with cisplatin–etoposide-based regimens or carboplatin–paclitaxel. No statistically significant difference in disease-free survival was detected between the treatment groups. However, distinct toxicity patterns were observed: peripheral neuropathy was more frequent with carboplatin–paclitaxel, nephrotoxicity occurred only in the cisplatin-based group, and pulmonary toxicity was observed only among patients exposed to bleomycin. Given the retrospective design, small sample size, and potential for treatment-selection bias, these findings should be considered exploratory and should not be interpreted as evidence of equivalent efficacy or superiority of either regimen.

## 1. Introduction

Adult granulosa cell tumors (AGCTs) are rare ovarian sex cord-stromal neoplasms characterized by an indolent clinical course, generally favorable long-term survival, and a persistent risk of late recurrence [1]. Given that relapse can occur many years after initial treatment, long-term surveillance is essential. Surgery remains the primary treatment modality; however, the role of adjuvant chemotherapy following primary resection remains uncertain, especially in patients with early stage disease [2]. Because AGCTs are rare, the evidence base supporting postoperative systemic treatment remains limited and derives mainly from retrospective series, mixed-sex cord-stromal tumor cohorts, and expert consensus rather than prospective randomized trials [3,4].

Current clinical guidelines generally recommend adjuvant chemotherapy only for selected patients with higher-risk features, such as stage IC2/IC3 disease, advanced-stage tumors, residual disease, or other adverse clinicopathological characteristics [5]. However, the available data remain inconsistent, and several retrospective studies in stage I disease have failed to demonstrate a clear survival benefit from adjuvant chemotherapy [6,7,8]. This uncertainty is clinically important because most patients with AGCTs present with stage I disease and often achieve excellent long-term outcomes with surgery alone. In this context, treatment-related toxicity constitutes a critical factor in postoperative decision-making.

Traditionally, bleomycin–etoposide–cisplatin (BEP), or etoposide–cisplatin (EP) in selected cases, has been widely used to manage AGCTs [9]. More recently, carboplatin–paclitaxel (CP) has been increasingly adopted, especially for older patients and those at elevated risk of cisplatin- or bleomycin-related toxicity [10]. However, comparative data between these regimens in the adjuvant setting are extremely limited. Recent studies continue to underscore uncertainty regarding both the role of chemotherapy and the optimal platinum-based regimen in AGCTs [10,11].

Given the lack of an established standard adjuvant regimen for AGCTs, additional data are required to clarify the balance between treatment burden and potential clinical benefit. This multicenter retrospective study compared BEP/EP and CP as adjuvant treatment strategies in patients with AGCTs, focusing on treatment completion, toxicity profiles, tolerability, and recurrence outcomes in a cohort predominantly comprising patients with stage I disease.

## 2. Materials and Methods

### 2.1. Study Design and Patient Population

**Figure 1:**
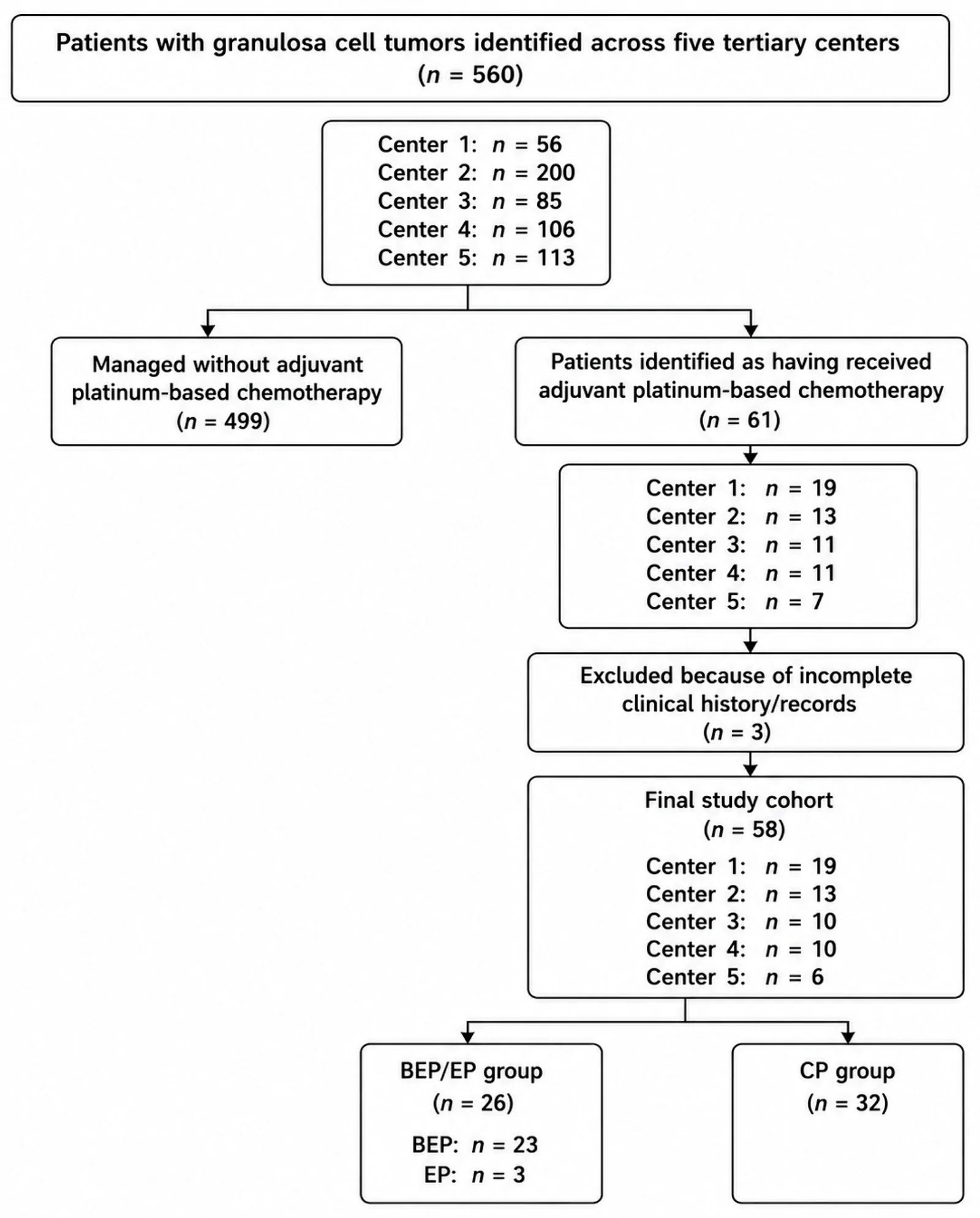
Patient-selection flow diagram of the multicenter retrospective cohort.

This was a retrospective, multicenter, nonrandomized comparative cohort study. Both treatment groups were identified retrospectively from institutional medical records; no patients were prospectively assigned to BEP/EP or CP, and treatment allocation was not protocol-driven. The study included patients diagnosed with AGCT who underwent primary surgery and received at least one cycle of adjuvant chemotherapy at five tertiary oncology centers in Türkiye between January 2010 and December 2024. Given the rarity of AGCTs, a multicenter design was adopted to increase sample size and improve the representativeness of real-world clinical practice. A total of 58 patients were included in the analysis. Eligible patients had histologically confirmed AGCT, FIGO stage I–III disease according to the 2014 International Federation of Gynecology and Obstetrics staging system, and had undergone primary surgery followed by adjuvant platinum-based chemotherapy [12]. A total of 560 patients with granulosa cell tumors were initially identified across the five participating tertiary centers. Following center-level screening, 61 patients who had received adjuvant platinum-based chemotherapy were assessed for eligibility. Three patients were excluded because of incomplete clinical history and insufficient medical-record data, leaving 58 patients in the final study cohort (Figure 1).

Patients received either BEP/EP or CP according to institutional practice patterns and physician discretion. Treatment selection was not protocol-driven and reflected real-world clinical decision-making based on individual patient characteristics, including age, comorbidities, fertility considerations, performance status, pulmonary risk factors, and anticipated treatment tolerability. Because the specific rationale for selecting BEP/EP versus CP was not systematically documented in the retrospective records, individual-level reasons for regimen selection could not be reliably reconstructed for every patient. Accordingly, BEP and EP were analyzed together as a single cisplatin–etoposide-based regimen group, with bleomycin omission occurring at the treating physician’s discretion in selected patients. Clinical, pathological, treatment-related, toxicity, and follow-up data were retrospectively collected from institutional medical records and electronic hospital databases. The study population was defined by receipt of postoperative platinum-based chemotherapy rather than by FIGO stage; therefore, patients with stage I disease were included if they had received adjuvant chemotherapy in routine clinical practice.

Fertility-sparing surgery was performed in selected younger patients according to reproductive preferences, disease extent, and the treating gynecologic oncologist’s discretion. Because fertility intentions and reproductive counseling were not prospectively standardized or uniformly documented across centers, the specific reproductive rationale for fertility preservation could not be reconstructed for every patient.

### 2.2. Variable Definitions

For statistical analyses, menopausal status was grouped as premenopausal/perimenopausal versus postmenopausal, and FIGO stage was categorized as stage I versus stages II–III.

FIGO stage was determined from the available surgical and pathologic records according to the 2014 International Federation of Gynecology and Obstetrics (FIGO) staging system [13]. For patients with stage I disease, substaging as IA, IC1, IC2, or IC3 was recorded when sufficient information was available; cases documented as stage IC without adequate information for further substaging were classified as IC not otherwise specified (IC-NOS). Complete surgical staging was defined as the performance of omentectomy, peritoneal washing cytology, and peritoneal biopsy. Preoperative and intraoperative tumor rupture were recorded separately. Surgical residual disease > 1 cm, as documented in the operative report, and postoperative radiologic residual disease were recorded when available. Missing surgical or pathologic information was not imputed. Fertility-sparing surgery was defined as preservation of the uterus and at least part of one ovary, including unilateral salpingo-oophorectomy with or without staging procedures.

Tumor size was categorized as ≤10 cm or >10 cm, based on prior studies indicating that larger tumors, particularly those exceeding 10–15 cm, may be associated with an increased risk of recurrence and poorer survival outcomes in AGCTs [12,14]. The mitotic index was categorized as ≤4 or >4 mitoses/mm^2^, consistent with thresholds used in previous studies to distinguish low and high mitotic activity [15]. Cytoreduction was classified as optimal when residual disease was ≤1 cm and suboptimal when residual disease was >1 cm, based on the operative report. Based on the available retrospective surgical records, complete gross resection could not be reliably distinguished from residual disease ≤ 1 cm in all patients. The decision to administer adjuvant chemotherapy and the choice of regimen were made by the treating physicians according to institutional practice and individual clinicopathologic considerations. As treatment indications were not prospectively standardized, the specific rationale for adjuvant chemotherapy could not be retrospectively established for every patient. Toxicities were categorized as hematologic or non-hematologic and graded as any grade or grade ≥ 3 according to the Common Terminology Criteria for Adverse Events (CTCAE) version 5.0 [16]. Toxicity data were retrospectively extracted from physicians’ progress notes and medical records, and CTCAE grades were assigned based on documented clinical information when not explicitly recorded.

### 2.3. Outcome Definition

The primary endpoint was disease-free survival (DFS), defined as the interval from primary surgery to recurrence or death from any cause, whichever occurred first. Secondary endpoints included treatment delivery outcomes, including completion of planned chemotherapy, treatment delay or interruption, permanent treatment discontinuation, and dose reduction, as well as treatment-related toxicity according to CTCAE version 5.0 [16].

### 2.4. Statistical Analysis

Categorical variables were summarized as frequencies and percentages and compared using the chi-square test or Fisher’s exact test, as appropriate. Continuous variables were summarized as mean ± standard deviation or median (interquartile range [IQR]), depending on distribution, and compared using the independent-samples *t*-test or Mann–Whitney U test, as appropriate. Missing data were handled using an available-case approach; therefore, the number of evaluable patients varied across variables depending on data availability. No imputation procedures were applied because of the retrospective design, limited sample size, and the risk of introducing additional uncertainty.

DFS was estimated using the Kaplan–Meier method and compared between groups using the log-rank test. Prognostic factors associated with DFS were evaluated using univariable Cox proportional hazards regression analysis. Variables considered clinically relevant and relevant to the primary study objective were entered into a multivariable Cox regression model to identify independent predictors of DFS. Because the number of DFS events was limited, multivariable modeling was restricted to a parsimonious set of covariates to minimize overfitting. Only clinically relevant variables and/or variables associated with DFS in univariable analysis were entered into the final Cox model. The proportional hazards assumption was assessed using time-dependent interactions between the model covariates and log-transformed survival time, with no evidence of violation. Treatment-emergent variables, such as dose reduction and toxicity, were not included in the multivariable model due to potential time-dependent bias. Hazard ratios (HRs) and 95% confidence intervals (CIs) were reported. A two-sided *p*-value < 0.05 was considered statistically significant. Statistical analyses were performed using IBM SPSS Statistics for Windows, version 27.0 (IBM Corp., Armonk, NY, USA).

## 3. Results

Among 58 patients analyzed, 26 received a cisplatin–etoposide-based regimen (23 received BEP and 3 received EP), and 32 received CP. Baseline characteristics were generally balanced between the treatment groups (Table 1). Patients in the CP group were significantly older (median 51.5 vs. 42.0 years; *p* = 0.031), whereas fertility-sparing surgery was more common in the cisplatin–etoposide-based regimen group (38.5% vs. 15.6%; *p* = 0.048). No significant differences were observed in menopausal status, comorbidities, preoperative ascites, stage, cytoreduction status, tumor size, mitotic index, or concomitant endometrial pathology (all *p*-values > 0.05). Overall, 39 patients (67.2%) had stage I disease. Among these, 13 (33.3%) had stage IA, 1 (2.6%) had stage IC1, 17 (43.6%) had stage IC2, and 5 (12.8%) had stage IC3 disease; in three patients (7.7%), stage IC was documented without sufficient information for further substaging. The corresponding distributions in the BEP/EP and CP groups were 6 versus 7 for stage IA, 0 versus 1 for IC1, 8 versus 9 for IC2, 3 versus 2 for IC3, and 1 versus 2 for IC not otherwise specified. Seven recurrences occurred among patients with stage I disease: six in the BEP/EP group and one in the CP group (Supplementary Table S1).

Among the 13 patients with stage IA disease, 6 (46.2%) had tumors > 10 cm, and 8 of 12 patients (66.7%) with available mitotic-index data had a mitotic index > 4 mitoses/mm^2^. At least one of these features was present in 9 patients (69.2%). Although the retrospective records could not reliably reconstruct the rationale for administering adjuvant chemotherapy for each patient, these clinicopathologic characteristics may have contributed to postoperative treatment decisions. Two recurrences occurred among patients with stage IA disease, both in the BEP/EP group. Complete surgical staging was performed in 4 of 13 patients (30.8%). Peritoneal washing was performed in 12 patients, with no positive cytology documented, and no pre- or intraoperative tumor rupture was recorded. Among patients with available data, none had >1 cm surgical residual disease or postoperative radiologic residual disease (Supplementary Table S2).

Among patients with stage IC disease, surgical and pathologic characteristics according to FIGO substage are detailed in Supplementary Table S3. No positive peritoneal cytology was identified among patients with IC1 or IC2 disease who underwent peritoneal washing, whereas positive cytology was documented in all five patients with IC3 disease. Intraoperative rupture was documented in the patient with IC1 disease, while preoperative rupture occurred predominantly in patients with IC2 and IC3 disease. Surgical residual disease >1 cm was uncommon, occurring in only one evaluable patient with IC2 disease. Recurrence occurred in 3 of 17 patients (17.6%) with IC2 and 2 of 5 patients (40.0%) with IC3 disease, with no recurrences observed in the IC1 or IC-NOS groups (Supplementary Table S3).

The three patients who received EP were individually reviewed. The retrospective medical records did not explicitly document the reason for omitting bleomycin. The patients were 61, 68, and 73 years old. Two had clinical characteristics that may have influenced the decision to omit bleomycin: one had ECOG performance status 2 with coronary artery disease and chronic obstructive pulmonary disease/asthma, and another had chronic kidney disease in addition to hypertension and diabetes. The third patient had ECOG performance status 0 and no documented pulmonary comorbidity; therefore, a specific reason for bleomycin omission could not be identified. Accordingly, EP selection was not attributed to a specific clinical indication when such an indication was not explicitly documented.

Regarding their clinical course, the 73-year-old patient completed the planned two cycles of EP without documented treatment delay, dose reduction, major treatment-related toxicity, or recurrence. The 68-year-old patient had four cycles planned but received only one cycle, with treatment interruption and dose modification in the setting of gastrointestinal toxicity; this patient subsequently developed recurrence. The 61-year-old patient received three of four planned cycles and experienced clinically significant hematologic toxicity, including grade 4 neutropenia, grade 3 febrile neutropenia, thrombocytopenia, and anemia, which contributed to treatment modification; no recurrence was documented during follow-up. Supplementary Table S4 summarizes the clinical characteristics, potential factors relevant to bleomycin omission, treatment delivery, toxicity, and recurrence status of the three patients treated with EP.

Baseline characteristics differed between treatment groups in several respects. Patients receiving CP were older (median, 51.5 vs. 42.0 years; *p* = 0.031) and more frequently underwent complete surgical staging (65.6% vs. 34.6%; *p* = 0.034), whereas fertility-sparing surgery was more frequent in the BEP/EP group (38.5% vs. 15.6%; *p* = 0.048). FIGO stage distribution and residual disease after surgery did not differ significantly between groups. Treatment patterns varied across participating centers, but treatment distribution did not differ significantly by calendar period (Supplementary Table S5).

**Figure 2:**
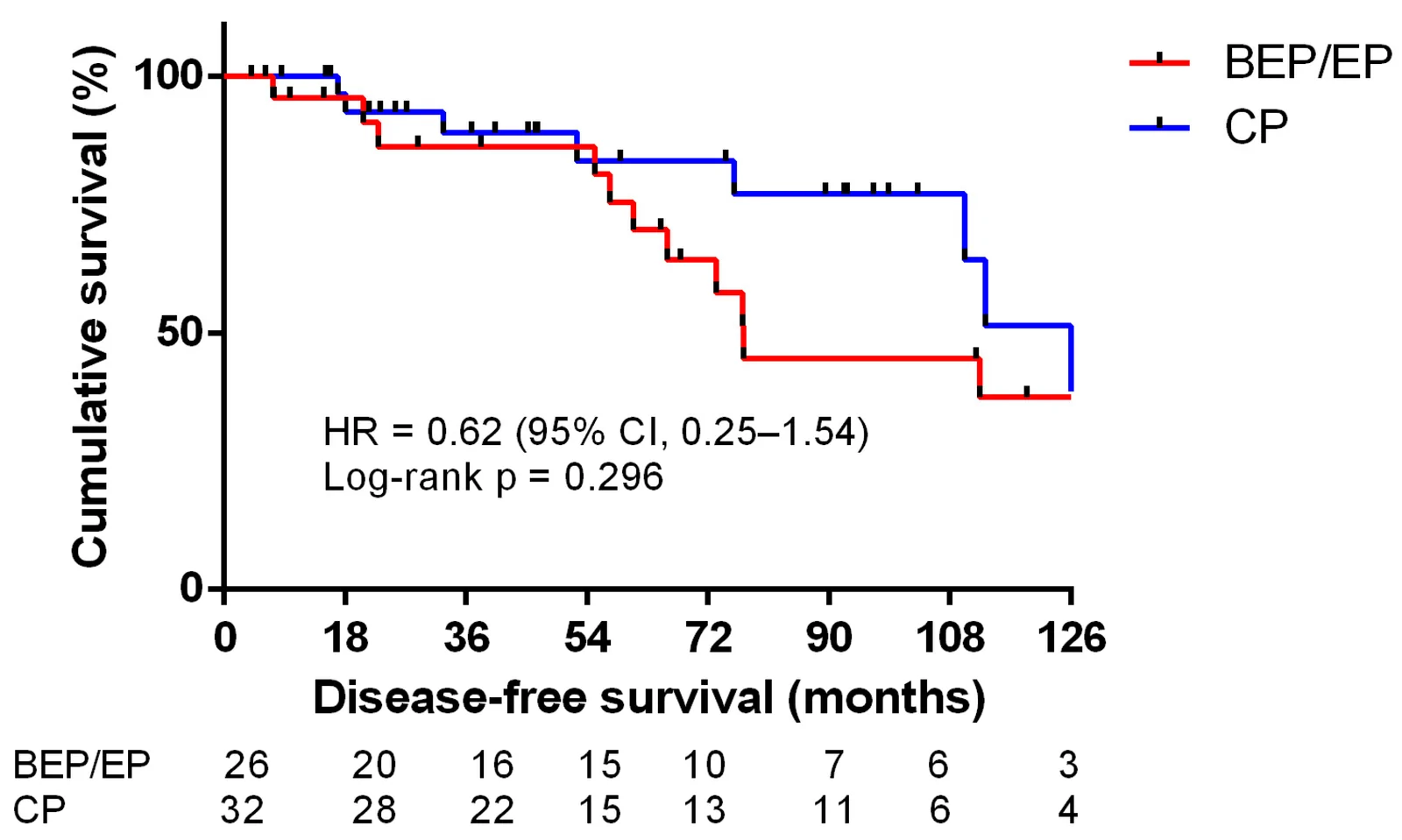
Kaplan–Meier curves for disease-free survival according to adjuvant chemotherapy regimen. No significant difference was observed between groups (log-rank *p* = 0.296). Numbers at risk are shown below the x-axis.

Kaplan–Meier analysis showed no statistically significant difference in DFS between treatment groups. Using the reverse Kaplan–Meier method, the median follow-up was 75.5 months (IQR, 42.3–119.9) for the overall cohort, 91.4 months (IQR, 66.9–123.5) in the BEP/EP group, and 58.9 months (IQR, 32.3–111.3) in the CP group. During follow-up, 14 recurrences (24.1%) were observed. When deaths without prior recurrence were also considered according to the prespecified DFS definition, a total of 19 DFS events occurred, including 11 in the BEP/EP group and 8 in the CP group. Median DFS was 113.3 months (95% CI, 94.3–132.3) in the overall cohort, 77.2 months (95% CI, 69.7–84.7) in the BEP/EP group, and 126.2 months (95% CI, 106.1–146.2) in the CP group. The estimated 5-year DFS rate was 75.5% (95% CI, 50.6–89.1%) in the BEP/EP group and 83.5% (95% CI, 60.8–93.7%) in the CP group, with no statistically significant difference between the groups (log-rank *p* = 0.296; Figure 2).

To address the potential limitation of using DFS in patients with residual or uncertain postoperative disease status, a sensitivity analysis was restricted to patients with documented postoperative residual disease ≤ 1 cm, excluding four patients with residual disease > 1 cm and nine patients with unknown residual-disease status. The restricted cohort included 45 patients (18 BEP/EP and 27 CP). Five DFS events occurred in the BEP/EP group and eight in the CP group. Median DFS was not reached in the BEP/EP group and was 113.3 months (95% CI, 93.0–133.6) in the CP group. The estimated 5-year DFS rates were 79.6% and 80.5%, respectively, with no statistically significant difference between treatment groups (log-rank *p* = 0.941).

In a sensitivity analysis excluding the three patients who received EP, 23 patients treated with BEP were compared with 32 patients treated with CP. Nine DFS events occurred in the BEP group and eight in the CP group. Median DFS was 112.4 months (95% CI, 48.1–176.7) in the BEP group and 126.2 months (95% CI, 106.1–146.2) in the CP group. The estimated 5-year DFS rates were 72.0% and 83.5%, respectively. No statistically significant difference in DFS was observed between BEP and CP (log-rank *p* = 0.435). In univariable Cox regression, CP was associated with a numerically lower hazard of a DFS event compared with BEP, although the difference was not statistically significant (HR 0.68, 95% CI 0.26–1.79; *p* = 0.438).

In univariate Cox regression analysis, increasing age was associated with a higher risk of a DFS event (HR 1.038 per year, 95% CI 1.002–1.076; *p* = 0.039). No other clinicopathologic variable, including the adjuvant treatment regimen, was significantly associated with DFS. Given the limited number of DFS events (*n* = 19), the multivariable model was deliberately restricted to age and adjuvant treatment regimen to minimize overfitting. In this exploratory model, increasing age remained independently associated with a higher risk of a DFS event (adjusted HR 1.046 per year, 95% CI 1.011–1.082; *p* = 0.010), whereas CP versus BEP/EP was associated with a numerically lower hazard that did not reach statistical significance (adjusted HR 0.426, 95% CI 0.167–1.083; *p* = 0.073). No evidence of violation of the proportional hazards assumption was identified for age (*p* = 0.909) or treatment regimen (*p* = 0.754). Detailed univariate and multivariable Cox regression analyses are provided in Supplementary Table S6.

Table 2 summarizes treatment delivery outcomes. In the cisplatin–etoposide-based regimen group, the median number of planned cycles was 3 (IQR, 3–4), whereas in the CP group, it was 6 (IQR, 6–6). Completion of all planned treatment was numerically more frequent with the CP regimen (84.4% vs. 69.2%; *p* = 0.17). Treatment delays or interruptions occurred in 38.5% of patients receiving the cisplatin–etoposide-based regimen and in 34.4% of patients receiving CP (*p* = 0.75). Permanent treatment discontinuation was observed in 30.8% and 15.6% of patients, respectively (*p* = 0.17). Dose reductions occurred in 19.2% of the cisplatin–etoposide-based regimen group and 28.1% of the CP group (*p* = 0.43). Among patients requiring dose reduction, hematologic toxicities were the primary reasons, most commonly neutropenia, febrile neutropenia, and thrombocytopenia. Non-hematologic toxicities, including peripheral neurotoxicity, nephrotoxicity, gastrointestinal toxicity, and pulmonary toxicity, were less frequent contributors (Supplementary Table S7).

**Figure 3:**
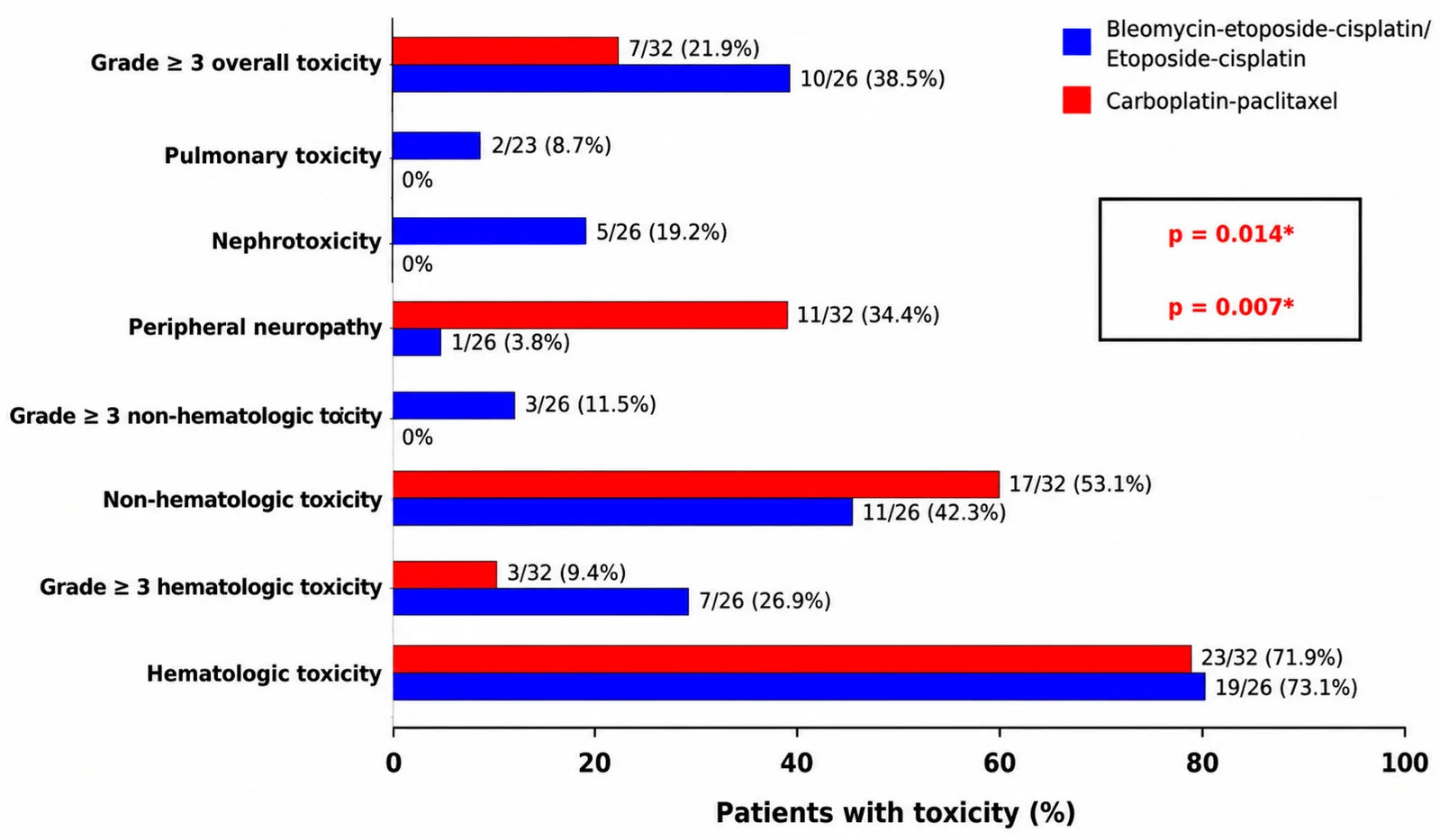
Treatment-related toxicities according to the adjuvant chemotherapy regimen. Values are presented as *n*/*N* (%). Only statistically significant *p*-values are displayed. * Fisher’s exact test.

Treatment-related toxicities are summarized in Figure 3. Hematologic toxicity rates were comparable between groups, with no significant differences in neutropenia, febrile neutropenia, thrombocytopenia, or anemia (all *p*-values > 0.05). Although grade ≥ 3 hematologic toxicity was numerically more frequent in the cisplatin–etoposide-based regimen group (26.9% vs. 9.4%), this did not reach statistical significance (*p* = 0.10). Regarding non-hematologic adverse events, peripheral neuropathy occurred significantly more frequently with CP (34.4% vs. 3.8%; *p* = 0.007), whereas nephrotoxicity was observed exclusively in the cisplatin-based regimen (19.2% vs. 0%; *p* = 0.014). Furthermore, grade ≥ 3 non-hematologic toxicities occurred only in the BEP/EP group, although this difference was not statistically significant. Pulmonary toxicity was observed in 2 of 23 patients exposed to bleomycin (8.7%) and was not observed among patients receiving EP or CP. Gastrointestinal toxicity rates were similar between the treatment groups.

Secondary malignancies during follow-up were rare and heterogeneous, comprising two endometrial neoplasms, one acute myeloid leukemia (AML), one meningioma, and one Sertoli–Leydig cell tumor. Notably, the only case of AML developed in a 42-year-old patient with FIGO stage IIIC disease after completion of four cycles of BEP.

## 4. Discussion

In interpreting the present findings, the distinct toxicity profiles observed with the two platinum-based regimens are consistent with their established adverse-effect spectra in gynecologic oncology. Specifically, peripheral neuropathy occurred more frequently with CP, consistent with the recognized neurotoxicity of paclitaxel-based chemotherapy [17,18]. Conversely, nephrotoxicity occurred only in the cisplatin–etoposide-based group, whereas pulmonary toxicity occurred only among patients exposed to bleomycin, consistent with the known renal toxicity of cisplatin and the pulmonary toxicity of bleomycin-containing regimens [19,20].

In our cohort, however, assessment of pulmonary toxicity was limited to clinically symptomatic events documented retrospectively, and serial pulmonary function testing, including DLCO monitoring, was not standardized across centers; therefore, subclinical toxicity may have been underrecognized. Importantly, one patient in the BEP/EP group developed secondary AML, a rare but recognized complication of etoposide-containing chemotherapy [21]. However, detailed latency, cumulative etoposide exposure, prior chemotherapy or radiotherapy exposure, and hematologic/cytogenetic information were not available in the retrospective records; therefore, a causal relationship with etoposide-containing chemotherapy cannot be established.

Our findings should be interpreted in the context of the limited comparative literature available for AGCTs and other sex cord-stromal tumors. Brown et al. previously suggested that taxane-based regimens may be associated with lower toxicity than BEP, supporting further evaluation of platinum–taxane combinations [22]. More recently, the randomized NRG/GOG phase II trial provided the highest-level comparative evidence available for platinum-based chemotherapy in ovarian sex cord-stromal tumors. In that trial, CP was associated with fewer grade ≥ 3 adverse events than BEP; however, it did not meet the prespecified non-inferiority criterion [10]. Importantly, the NRG/GOG population included a broader spectrum of sex cord-stromal tumors in newly diagnosed advanced-stage or recurrent chemotherapy-naïve settings and was not designed specifically to evaluate postoperative adjuvant chemotherapy in AGCTs.

Our study addresses a distinct clinical setting by providing multicenter real-world data specifically on patients with AGCT receiving postoperative adjuvant chemotherapy, with the cohort predominantly comprising stage I disease. Rather than establishing comparative efficacy or non-inferiority between regimens, our findings characterize treatment delivery, DFS outcomes, and regimen-specific toxicity patterns in contemporary adjuvant practice. The absence of a statistically significant difference in DFS should not be interpreted as evidence of equivalent efficacy. Instead, our findings complement the randomized NRG/GOG data by providing additional information on the real-world use and tolerability of these regimens in the adjuvant AGCT setting. This interpretation is consistent with the Cochrane review emphasizing the limited and predominantly retrospective evidence base in AGCTs [23].

The survival results of our study are consistent with a growing body of retrospective literature questioning the benefit of adjuvant chemotherapy in AGCTs, particularly in early stage disease. In the SALOME study and other retrospective series, adjuvant chemotherapy was not associated with improved DFS or overall survival in stage I–II AGCTs, whereas surgical completeness consistently emerged as a major prognostic factor [6,10,11,24,25,26]. Similarly, a recent systematic review and meta-analysis of stage IC granulosa cell tumors found no significant improvement in recurrence, DFS, or overall survival with adjuvant chemotherapy and did not demonstrate superiority of a cisplatin–etoposide-based regimen over taxane–platinum combinations [27]. These findings are particularly relevant to our cohort, which consisted predominantly of stage I patients.

Reproductive considerations are particularly relevant when adjuvant chemotherapy is contemplated in younger patients undergoing fertility-sparing surgery. Although preservation of the uterus and ovarian tissue may maintain the possibility of future fertility, subsequent cytotoxic chemotherapy can impair ovarian reserve and reproductive potential. The risk of treatment-related gonadotoxicity depends on the chemotherapy regimen, cumulative exposure, and patient age, and should therefore be incorporated into treatment counseling. In women with future reproductive plans, the uncertain benefit of adjuvant chemotherapy in early stage AGCT should be weighed not only against acute and long-term systemic toxicities but also against potential effects on fertility. Current ASCO guidance recommends that reproductive risks be discussed before cancer-directed therapy and that patients interested in or uncertain about fertility preservation be referred promptly to reproductive specialists for consideration of established fertility-preservation strategies [28].

Notably, adjuvant chemotherapy was also administered to a subset of patients with stage IA disease in our cohort, reflecting the heterogeneity of real-world clinical decision-making in AGCTs. Given the absence of definitive prospective data and the persistent concern for late recurrence, physicians may consider additional clinicopathologic features beyond stage alone, including tumor size, mitotic activity, intraoperative findings, fertility considerations, or perceived recurrence risk when recommending postoperative treatment. In this regard, our cohort may better reflect contemporary real-world practice patterns than highly selected trial populations.

Beyond cytotoxic chemotherapy, other treatment modalities may have a role in selected patients with recurrent or advanced AGCT. Radiotherapy can provide meaningful local control in selected patients with unresectable localized or recurrent disease, with occasional durable responses reported in retrospective series [29]. Hormonal therapy is also a relevant option in recurrent disease. In the prospective PARAGON/ANZGOG 0903 trial, anastrozole produced a 12-week clinical benefit rate of 78.9% in patients with recurrent or metastatic hormone receptor-positive granulosa cell tumors, although the objective response rate was modest [30]. More recently, leuprolide acetate-based therapy was associated with a 6-month clinical benefit rate of 66% and a median progression-free survival of 10.3 months in recurrent granulosa cell tumors [31]. Antiangiogenic therapy has also demonstrated activity; in a Gynecologic Oncology Group phase II trial, single-agent bevacizumab achieved a response rate of 16.7%, with stable disease in 77.8% of patients and a median progression-free survival of 9.3 months in recurrent sex cord-stromal tumors [32]. These approaches may therefore represent treatment options for selected patients with recurrent or advanced disease, although the supporting evidence remains limited and should not be extrapolated to the postoperative adjuvant setting.

The indolent natural history of AGCTs further complicates the interpretation of adjuvant treatment efficacy. Although platinum-based chemotherapy demonstrates activity in recurrent or measurable disease [33,34], this does not necessarily translate into the eradication of microscopic residual disease after primary surgery. In our study, recurrence was observed in only approximately one-quarter of patients despite long-term follow-up exceeding 7 years, underscoring the prolonged clinical course of AGCTs. Given the established prognostic importance of surgical completeness in AGCT, the limitations of retrospective residual-disease documentation should be considered when interpreting our survival findings. From a clinical perspective, these findings underscore the uncertainty surrounding postoperative treatment selection, particularly in completely resected early stage disease, where the balance between uncertain survival benefit and treatment-related toxicity remains clinically relevant.

This study has several limitations that should be considered when interpreting the findings. First, its retrospective, nonrandomized design introduces potential for selection bias and residual confounding. In addition, the limited number of DFS events reduced the robustness of multivariable analyses despite the use of a parsimonious model; therefore, the adjusted survival estimates should be interpreted with caution. The study was also not powered to detect modest differences in efficacy between regimens, and because most patients had stage I disease, the findings primarily reflect a predominantly early stage cohort.

An additional limitation is the heterogeneity and incompleteness of retrospective surgical and pathologic documentation. Although FIGO stage I substaging, surgical staging procedures, tumor rupture, and residual disease were reconstructed whenever supported by the available records, complete information was not available for all patients. In particular, the specific rationale for administering adjuvant chemotherapy to patients with stage IA disease could not be established on an individual-patient basis. Therefore, clinicopathologic features observed in these patients, including large tumor size and high mitotic activity, should be regarded as potential factors that may have influenced treatment selection rather than documented indications for chemotherapy.

Follow-up duration also differed between treatment groups, with a longer median follow-up in the BEP/EP group than in the CP group. This imbalance may reflect temporal and institutional differences in treatment selection and should be considered when interpreting comparative survival outcomes. Treatment allocation was nonrandom and varied according to patient and institutional characteristics, including age, fertility-sparing surgery, completeness of surgical staging, and institutional practice patterns. These imbalances, together with the longer follow-up in the BEP/EP group, indicate the potential for confounding by indication and treatment-selection bias.

Toxicity assessment was limited by the retrospective design and reliance on medical records rather than prospective, standardized toxicity monitoring. Accordingly, adverse-event frequencies and grades should be considered descriptive and exploratory, and underreporting of lower-grade or subclinical toxicities cannot be excluded. In particular, pulmonary toxicity assessment was based on clinically documented symptomatic events, while serial pulmonary function testing and lung diffusing-capacity monitoring were not standardized across centers. Another limitation is the heterogeneity of the BEP/EP group, as not all patients received bleomycin; accordingly, this cohort should be interpreted more broadly as representing a cisplatin–etoposide-based approach. Finally, given the rarity and prolonged clinical course of AGCTs, our findings should be considered hypothesis-generating and warrant validation in larger collaborative datasets.

## 5. Conclusions

In conclusion, no statistically significant difference in DFS was detected between BEP/EP and CP in this multicenter retrospective cohort, while distinct toxicity patterns were observed between the regimens. Given the small sample size, limited number of DFS events, nonrandom treatment allocation, and potential for substantial selection bias and residual confounding, these findings should not be interpreted as evidence of equivalent efficacy or as establishing the superiority of either regimen. The results should therefore be considered exploratory and hypothesis-generating. Larger collaborative studies with improved risk stratification are needed to better define the role and optimal choice of postoperative chemotherapy in AGCTs.

## Figures and Tables

**Table 1:** Baseline clinicopathologic characteristics according to adjuvant treatment regimen.

Variable	Overall (*n* = 58)	BEP/EP (*n* = 26)	CP (*n* = 32)	*p* Value
Age at diagnosis, years, median (IQR)	47 (38.8–58.3)	42.0 (32.0–50.0)	51.5 (42.8–59.5)	0.031
Menopausal status				0.10
Premenopausal/perimenopausal	31 (53.4%)	17 (65.4%)	14 (43.8%)	
Postmenopausal	27 (46.6%)	9 (34.6%)	18 (56.2%)	
Comorbidity				0.27
No	31 (53.4%)	16 (61.5%)	15 (46.9%)	
Yes	27 (46.6%)	10 (38.5%)	17 (53.1%)	
Preoperative ascites				0.83
Absent	38 (73.1%)	15 (71.4%)	23 (74.2%)	
Present	14 (26.9%)	6 (28.6%)	8 (25.8%)	
Stage group				0.96
Stage I	39 (67.2%)	18 (69.2%)	21 (65.6%)	
Stage II–III	19 (32.8%)	8 (30.8%)	11 (34.4%)	
Fertility-sparing surgery	15 (25.9%)	10 (38.5%)	5 (15.6%)	0.048
Complete surgical staging	30 (51.7%)	9 (34.6%)	21 (65.6%)	0.034
Residual disease after surgery				
≤1 cm	45 (77.6%)	18 (69.2%)	27 (84.4%)	0.301 *
>1 cm	4 (6.9%)	3 (11.5%)	1 (3.1%)	
Unknown	9 (15.5%)	5 (19.2%)	4 (12.5%)	
Tumor size				0.19
≤10 cm	21 (36.2%)	7 (26.9%)	14 (43.8%)	
>10 cm	37 (63.8%)	19 (73.1%)	18 (56.2%)	
Mitotic index				0.66
≤4 mitoses/mm^2^	14 (30.4%)	6 (27.3%)	8 (33.3%)	
>4 mitoses/mm^2^	32 (69.6%)	16 (72.7%)	16 (66.7%)	
Concomitant endometrial pathology				0.087
Absent	33 (56.9%)	18 (69.2%)	15 (46.9%)	
Present	25 (43.1%)	8 (30.8%)	17 (53.1%)	

Footnote: Abbreviations: IQR, interquartile range. Categorical variables are presented as *n* (%), and continuous variables as median (interquartile range [IQR]). Comparisons between treatment groups were performed using the chi-square test, Fisher’s exact test, or Mann–Whitney U test, as appropriate. Preoperative ascites data were available for 52 patients, and mitotic index data were available for 46 patients. Staging was based on the 2014 International Federation of Gynecology and Obstetrics (FIGO) classification. * The comparison of residual disease after surgery was performed among patients with available data, excluding patients with unknown residual disease status.

**Table 2:** Treatment delivery and treatment completion outcomes according to the adjuvant chemotherapy regimen.

Variable	BEP/EP (*n* = 26)	CP (*n* = 32)	*p*-Value
Planned cycles, median (IQR)	3 (3–4)	6 (6–6)	— ^&^
Completed cycles, median (IQR)	3 (2–3)	6 (6–6)	— ^&^
Completed planned treatment, *n* (%)	18 (69.2)	27 (84.4)	0.17
Cycle delay/interruption, *n* (%)	10 (38.5)	11 (34.4)	0.75
Permanent treatment discontinuation, *n* (%)	8 (30.8)	5 (15.6)	0.17
Dose reduction, *n* (%)	5 (19.2)	9 (28.1)	0.43

Footnote: Continuous variables are presented as median (IQR) and were compared using the Mann–Whitney U test. Categorical variables are presented as *n* (%) and were compared using the chi-square test or Fisher’s exact test, as appropriate. ^&^  Planned and completed cycle numbers were descriptive treatment-delivery measures and were not formally compared because treatment protocols differed between regimens.

## Data Availability

The data supporting the findings of this study are available from the corresponding author upon reasonable request. The data are not publicly available because they contain information that could compromise the privacy of research participants and are subject to institutional data protection policies.

## Supplementary Materials

The following supporting information can be downloaded at: https://www.mdpi.com/article/10.3390/cancers18182910/s1. Table S1: FIGO stage I substaging and recurrences by treatment group; Table S2: Clinicopathologic and surgical characteristics of patients with FIGO stage IA adult granulosa cell tumor receiving adjuvant chemotherapy; Table S3: Surgical and pathologic characteristics of patients with FIGO stage IC adult granulosa cell tumor according to substage; Table S4: Clinical characteristics and treatment course of patients receiving etoposide–cisplatin; Table S5: Distribution of adjuvant chemotherapy according to participating center and calendar period; Table S6: Univariate and multivariable Cox regression analyses for disease-free survival; Table S7: Toxicities leading to treatment modification according to adjuvant chemotherapy regimen.

## Abbreviations

AGCT	Adult granulosa cell tumor
AML	Acute myeloid leukemia
ANZGOG	Australia New Zealand Gynaecological Oncology Group
ASCO	American Society of Clinical Oncology
BEP	Bleomycin–etoposide–cisplatin
EP	Etoposide–cisplatin
BEP/EP	Bleomycin–etoposide–cisplatin/etoposide–cisplatin
CP	Carboplatin–paclitaxel
CI	Confidence interval
CTCAE	Common Terminology Criteria for Adverse Events
DFS	Disease-free survival
DLCO	Diffusing capacity of the lung for carbon monoxide
ECOG	Eastern Cooperative Oncology Group
FIGO	International Federation of Gynecology and Obstetrics
HR	Hazard ratio
IC-NOS	Stage IC not otherwise specified
IQR	Interquartile range
NRG/GOG	NRG Oncology/Gynecologic Oncology Group
