# Supplementary material for: Treatment Outcomes and Toxicity of Cisplatin–Etoposide-Based Regimens and Carboplatin–Paclitaxel as Adjuvant Chemotherapy for Ovarian Adult Granulosa Cell Tumors: A Multicenter Retrospective Cohort Study
Source: Cancers (Basel). 2026 Sep 9;18(18):2910. doi: 10.3390/cancers18182910 (PMC13604064; doi:10.3390/cancers18182910)
Supplement: Supplementary file 1 [file cancers-18-02910-s001.zip › cancers-4507480-supplementary.pdf]

Supplementary Table S1. FIGO stage I substaging and recurrences by treatment group

FIGO substage	BEP/EP (n=18)	CP (n=21)	Recurrences, BEP/EP	Recurrences, CP
IA	6 (33.3%)	7 (33.3%)	2/6 (33.3%)	0/7 (0%)
IC1	0 (0%)	1 (4.8%)	0	0/1 (0%)
IC2	8 (44.4%)	9 (42.9%)	3/8 (37.5%)	0/9 (0%)
IC3	3 (16.7%)	2 (9.5%)	1/3 (33.3%)	1/2 (50.0%)
IC, NOS*	1 (5.6%)	2 (9.5%)	0/1 (0%)	0/2 (0%)
Total	18 (100%)	21 (100%)	6/18 (33.3%)	1/21 (4.8%)

Footnote: *IC, NOS: stage IC disease documented without sufficient information for further substaging.

Supplementary Table S2. Clinicopathologic and surgical characteristics of patients with FIGO stage IA adult granulosa cell tumor receiving adjuvant chemotherapy

**Characteristic**	**Overall (n=13)**	**BEP/EP (n=6)**	**CP (n=7)**
Age, median (IQR), years	56 (46–61)	48 (35.5–58.3)	58 (53.5–63)
Tumor size, median (IQR), cm	9 (5–15)	9.5 (4.6–18.5)	9 (5.5–13)
Tumor size >10 cm	6 (46.2%)	3 (50.0%)	3 (42.9%)
High mitotic activity (>4 mitoses/mm^2)*	8/12 (66.7%)	3/5 (60.0%)	5/7 (71.4%)
Fertility-sparing surgery	3 (23.1%)	2 (33.3%)	1 (14.3%)
Complete surgical staging†	4 (30.8%)	1 (16.7%)	3 (42.9%)
Peritoneal washing performed	12 (92.3%)	5 (83.3%)	7 (100%)
Positive peritoneal cytology‡	0/12 (0%)	0/5 (0%)	0/7 (0%)
Pre-/intraoperative tumor rupture	0 (0%)	0 (0%)	0 (0%)
Residual disease >1 cm§	0/8 (0%)	0/4 (0%)	0/4 (0%)
Radiologic residual disease¶	0/7 (0%)	0/4 (0%)	0/3 (0%)
Recurrence	2 (15.4%)	2 (33.3%)	0 (0%)

Footnote: *Mitotic-index data were available for 12 of 13 patients.
† Complete surgical staging was defined as the performance of omentectomy, peritoneal washing cytology, and peritoneal biopsy.
‡ Peritoneal cytology results were available for the 12 patients who underwent peritoneal washing.
§ Surgical residual-disease information was available for 8 patients. Optimal cytoreduction was defined as residual disease ≤1 cm, whereas residual disease >1 cm was classified as suboptimal cytoreduction.
¶ Postoperative radiologic residual-disease information was available for 7 patients.

Supplementary Table S3. Surgical and pathologic characteristics of patients with FIGO stage IC adult granulosa cell tumor according to substage

**Characteristic**	**IC1 (n=1)**	**IC2 (n=17)**	**IC3 (n=5)**	**IC-NOS (n=3)**
Complete surgical staging	1 (100%)	10 (58.8%)	2 (40.0%)	1 (33.3%)
Peritoneal washing performed	1 (100%)	14 (82.4%)	5 (100%)	2 (66.7%)
Positive peritoneal cytology*	0/1 (0%)	0/14 (0%)	5/5 (100%)	NA
No tumor rupture	0	7 (41.2%)	1 (20.0%)	3 (100%)
Intraoperative tumor rupture	1 (100%)	1 (5.9%)	1 (20.0%)	0
Preoperative tumor rupture	0	9 (52.9%)	3 (60.0%)	0
Residual disease >1 cm†	0/1 (0%)	1/15 (6.7%)	0/5 (0%)	0/2 (0%)
Postoperative radiologic residual disease‡	0/1 (0%)	1/15 (6.7%)	1/5 (20.0%)	0/2 (0%)
Recurrence	0/1 (0%)	3/17 (17.6%)	2/5 (40.0%)	0/3 (0%)

Footnote: *Cytology results are presented among patients who underwent peritoneal washing. Cytology results were unavailable for patients classified as IC-NOS.
† Surgical residual-disease information was available for 1, 15, 5, and 2 patients with IC1, IC2, IC3, and IC-NOS disease, respectively.
‡ Postoperative radiologic residual-disease information was available for 1, 15, 5, and 2 patients with IC1, IC2, IC3, and IC-NOS disease, respectively.

Supplementary Table S4. Clinical characteristics and treatment course of patients receiving etoposide–cisplatin

**Characteristic**	**Patient 1**	**Patient 2**	**Patient 3**
Age, years	73	68	61
ECOG performance status	2	1	0
FIGO stage	IA	IC2	IA
Relevant comorbidities	Hypertension, coronary artery disease, COPD/asthma, hyperlipidemia	Hypertension, diabetes mellitus, chronic kidney disease	Hypertension, diabetes mellitus, obesity
Potential clinical factors relevant to bleomycin omission	Advanced age, ECOG 2, and cardiopulmonary comorbidity	Age and renal comorbidity	No specific factor could be identified from the available records

Characteristic	Patient 1	Patient 2	Patient 3
Documented reason for bleomycin omission	Not available	Not available	Not available
Planned/completed EP cycles	2/2	4/1	4/3
Treatment modification / major toxicity	None documented	Treatment interruption/modification associated with gastrointestinal toxicity	Treatment modification associated with significant hematologic toxicity, including febrile neutropenia
Recurrence	No	Yes	No

Footnote: The specific reason for omitting bleomycin was not explicitly documented in the retrospective medical records. Therefore, the clinical characteristics shown above are presented as potential factors that may have influenced treatment selection and should not be interpreted as documented indications for EP. COPD, chronic obstructive pulmonary disease; ECOG, Eastern Cooperative Oncology Group; EP, etoposide–cisplatin.

Supplementary Table S5. Distribution of adjuvant chemotherapy according to participating center and calendar period

Variable	BEP/EP, n (%)	CP, n (%)	Total, n
**Participating center**			
Center 1	14 (73.7)	5 (26.3)	19
Center 2	4 (30.8)	9 (69.2)	13
Center 3	2 (20.0)	8 (80.0)	10
Center 4	4 (40.0)	6 (60.0)	10
Center 5	2 (33.3)	4 (66.7)	6
**Calendar period**			
2010–2016	12 (46.2)	11 (34.4)	23
2017–2024	14 (53.8)	21 (65.6)	35
**Total cohort**	**26 (44.8)**	**32 (55.2)**	**58**

Footnote: Percentages for participating centers represent the distribution of treatment regimens within each center, whereas percentages for calendar periods represent the distribution of calendar periods within each treatment group. The distribution of treatment according to calendar period did not differ significantly between groups (p=0.425).

Supplementary Table S6. Univariate and multivariable Cox regression analyses for disease-free survival

Variable	Univariate HR (95% CI)	p-value	Adjusted HR (95% CI)	p-value
Age, per year	1.038 (1.002–1.076)	0.039	1.046 (1.011–1.082)	0.010
Menopausal status	0.479 (0.191–1.202)	0.117	—	—
Comorbidity	0.606 (0.243–1.512)	0.283	—	—
Preoperative ascites	2.524 (0.946–6.736)	0.064	—	—
Stage group	0.849 (0.340–2.119)	0.726	—	—
Fertility-sparing surgery	3.438 (0.789–14.982)	0.100	—	—
Complete surgical staging	1.661 (0.645–4.278)	0.294	—	—
Postoperative residual disease (>1 cm vs ≤1 cm)	1.031 (0.133–8.021)	0.977	—	—
Tumor size group	1.312 (0.511–3.370)	0.572	—	—
Mitotic index group	0.742 (0.287–1.916)	0.537	—	—
Concomitant endometrial pathology	1.280 (0.519–3.155)	0.592	—	—
Adjuvant treatment (CP vs BEP/EP)	0.617 (0.247–1.540)	0.301	0.426 (0.167–1.083)	0.073

Footnote: HR, hazard ratio; CI, confidence interval; CP, carboplatin–paclitaxel; BEP/EP, bleomycin–etoposide–cisplatin/etoposide–cisplatin. Given the limited number of DFS events (n=19), the multivariable model was deliberately restricted to age and adjuvant treatment regimen to minimize overfitting. Adjusted estimates should be considered exploratory

Supplementary Table S7. Toxicities Leading to Treatment Modification According to Adjuvant Chemotherapy Regimen.

**Cause of dose modification**	**BEP/EP (n=26)**	**CP (n=32)**
Dose reduction, n (%)	5 (19.2)	9 (28.1)
Hematologic toxicity-related modification, n (%)	3 (11.5)	8 (25.0)
Neutropenia/febrile neutropenia	2 (7.7)	3 (9.4)
Thrombocytopenia	1 (3.8)	3 (9.4)

**Cause of dose modification**	**BEP/EP (n=26)**	**CP (n=32)**
Anemia	0	2 (6.3)
Non-hematologic toxicity-related modification, n (%)	3 (11.5)	1 (3.1)
Peripheral neurotoxicity	0	1 (3.1)
Nephrotoxicity	1 (3.8)	0
Gastrointestinal toxicity	1 (3.8)	0
Pulmonary toxicity	1 (3.8)	0

Footnote: Toxicity categories were not mutually exclusive; therefore, subgroup counts may exceed the total number of patients with dose reduction. Percentages were calculated using the total number of patients in each treatment group as the denominator.
